# Impact of Grassland Reseeding, Herbicide Spraying and Ploughing on Diversity and Abundance of Soil Arthropods

**DOI:** 10.3389/fpls.2016.01200

**Published:** 2016-08-09

**Authors:** Wei Liu, Junling Zhang, Stuart L. Norris, Philip J. Murray

**Affiliations:** ^1^Collaborative Innovation Center of Jiangxi Typical Trees Cultivation and Utilization, Jiangxi Agricultural UniversityNanchang, China; ^2^Sustainable Soil and Grassland Systems, Rothamsted ResearchOkehampton UK; ^3^College of Resources and Environmental Sciences, China Agricultural UniversityBeijing, China

**Keywords:** soil fauna community, herbicide, ploughing, reseeding, arthropods

## Abstract

In order to determine the interactive effect of reseeding, herbicide spraying and ploughing on soil fauna communities, we conducted a grassland reseeding experiment combined with pre-reseed management to examine how with the whole reseeding process affects soil faunal composition. Sampling occasions and exact treatments were as follows: (1) before chemical herbicide spray; (2) after spray but before ploughing; (3) after ploughing but before reseeding; and (4) after 1 year of recovery. Our results demonstrate that, Acari and Collembola were the two soil fauna taxa with the highest abundance and accounted for around 96% of the relative total abundance among the various managements. Herbicide application tended to increase soil invertebrate abundance. Conversely, subsequent ploughing significantly reduced soil invertebrate abundance and had an obvious negative effect on soil primary and secondary decomposers, which were mainly due to the variations of Acari (especially Oribatida) and Coleoptera group abundance. Moreover, reseeding also reduced the individual number of the groups mentioned above, and favored those predators with a larger body size and individual weight. After 1 year recovery, Collembola abundance recovered to the pre-treatment levels, while with Arthropod and Acari groups were still fluctuating.

## Introduction

The soil fauna account for a large part of the global biodiversity (Kremen et al., 1993) and play key roles in many ecosystems (García et al., 2010; Carrillo et al., 2011; Basset et al., 2012) because they directly or indirectly influence soil function (Diekötter et al., 2010). For example, approximately 90% of aboveground primary production, in terrestrial ecosystems, enters the belowground system (Gessner et al., 2010) where the soil biota undertake the decomposition and mineralization of soil organic matter (Bernard et al., 2012).

The perennial nature of grasslands means that interactions between the plant and the soil are crucial in regulating soil processes (Murray et al., 2012). The perenniality of grassland ecosystems implies that they generally have a relatively stable and permanent plant cover which provides a secure habitat for abundant and diverse soil invertebrate fauna that contribute to effective soil functioning. Alongside this, grasslands tend to have a high turnover of root and shoot material than in other ecosystems and this, together with animal inputs, results in a relatively high level of organic matter content. This in turn allows grassland to support numerous and diverse biota, important for soil function (Murray et al., 2012). Soil fauna within grazed grassland break down both the labile and recalcitrant plant compounds releasing the nutrients bound up within them, so that they can be exploited by the plant (Wardle, 1999) and the biogeochemical cycling continues (Wall et al., 2010).

Although grasslands generally provide a stable soil environment, agricultural grassland management practices such as reseeding can affect sward structure and plant species composition (Celaya et al., 2007; García et al., 2010) and, probably more importantly, the soil structure and habitat. Therefore, such interventions in these systems can have a knock-on effect on the associated soil fauna (Gibson et al., 1992; Dennis et al., 1998, 2001, 2008). The soil fauna has been shown to be sensitive to changes in soil conditions (Vasconcellos et al., 2013) and soil management practices can also have an effect on the energy channels (bacterial feeding channel and fungal feeding channel) and soil food webs (Doblas-Miranda et al., 2008; Maharning et al., 2009; Strickland and Rousk, 2010). For example, in cropping systems, conventional tillage is thought to promote the bacterial energy channel in the soil food web by the redistribution of plant residues within the soil during ploughing; in comparison, no tillage systems are thought to promote the fungal energy channel and the immobilization of plant nutrients (Hendrix et al., 1986). Types of tillage also had conflicting effects on soil arthropods, for example, Petersen (2002) showed that conventional ploughing reduced the collembolan population more than the non-inverting tillage does in upper soil stratum, while the two tillage treatments resulted in similar population changes for most collembolan species when take the whole soil horizon into considered. Studies involving herbicide application also had distinct effects. Lins et al. (2007) studied the effects of different herbicides on Collembola, and found that the use of atrazine and 2,4-D significantly decreased Collembola diversity but it depended on the handling time in a no-till soil preparation system. Nevertheless, Greenslade et al. (2010) found that herbicides have no significant effect on surface-active arthropods although Collembola were more affected than Formicidae in the short term. Some soil faunal groups (e.g., spiders, harvestmen) and some ground beetles are known to react strongly to such changes in microhabitat conditions and are subsequently often used as indicators of the effects of management practices (Hillyard and Sankey, 1989; Bell et al., 2001; Rainio and Niemelä, 2003). However, knowledge of such interactions occurring between the faunal community is limited, because the factors responsible for this high diversity of soil animals on small spatial scales are still not fully understood (Maraun et al., 2011). This applies especially to the high α-diversity, which implies the existence of a large number of niches in a very small area (Maraun et al., 2007), possibly because below-ground animal taxa are generalists that inhabit wide niches. In this study we determine the impact of perturbation (herbicide, ploughing), during grassland reseeding on the soil fauna and its subsequent recovery.

## Materials and Methods

### Study Site Description and Experimental Design

The study site was located at the North Wyke Farm Platform (NWFP) in the South West of England (50°46′55″N, 3°55′1″W) and is fully described in Orr et al. (2016). The vegetation is permanent grassland dominated by perennial ryegrass (*Lolium perenne* L.) and creeping bent (*Agrostis stolonifera* L.); the soil is classified as clayey pelo-stagnogley developed from located mainly under gentle low lying slopes (Hallsworth series, Harrod and Hogan, 2008). The main properties of the soil were organic carbon 3.7%, nitrogen 0.5% and phosphorus 0.1%, with a clay content of 38%, bulk density of 0.99 g cm^-3^ and a pH of 5.3 (Harrod and Hogan, 2008). Average annual rainfall at the site is 1085 mm (± 8.5), average air temperature is 9.8°C (± 0.65); yearly total sunlight is 1419 h (± 38) and average soil temperature is 10.9°C (± 0.77; Crotty et al., 2012).

**FIGURE 1:**
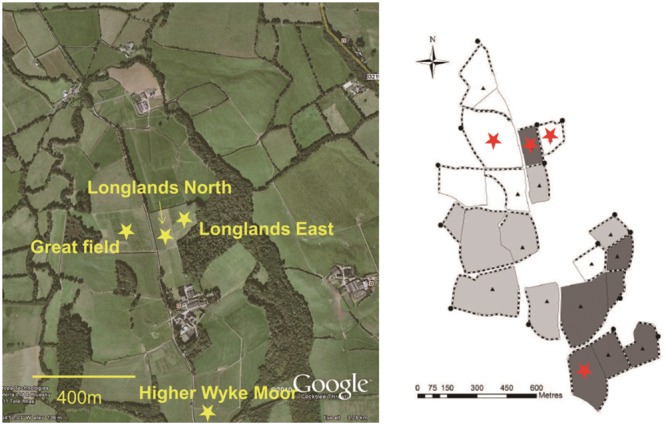
**Distribution of grassland management fields of the Rothamsted Research (North Wyke) in South-west UK.** Samples were collected from four grassland management field: Higher Wyke Moor (HWM), Longlands North (LN), Great Field (GF), and Longlands East (LE) (Provided by Prof. Philip J. Murray).

The main aim of this study was to determine the impact of perturbation, during grassland management (reseeding, herbicide, ploughing) on the soil faunal composition. Four field permanent grasslands were chosen (**Figure 1**) were due to be reseeded. Prior to reseeding all the fields were under the same management regime, receiving 200 kg N per annum. In June 2013 the were sprayed with glyphosate (Glyphosate 360, Dow Agrosciences, Hitchin, UK), 6 weeks later the fields were ploughed and a seedbed prepared with subsequent reseeding in August 2013.

### Soil Sampling

Soil samples were collected four times during the study: (1) before chemical herbicide spraying (designated S1, 26th June), (2) after spraying but before ploughing (designated S2, 3th July), (3) after ploughing but before reseeding (designated S3, 24th July), and (4) after 1 year of recovery (designated S4, 26th August, 2014). Intact soil cores (8 cm diameter, 10 cm deep; weighing on average 1.2 ± 0.02 kg, wet weight) were taken from the four fields. Overlaying the NWFP is a GPS defined 50 m grid and on each sampling occasion, six grid locations were randomly selected and a single soil core was collected at each point. Each individual core was stored within an individual Sun bag (Sigma-Aldrich, St. Louis, MO, USA). Post-extraction the invertebrates from each field were amalgamated to provide a single sample for each field for each sampling occasion. The soil characteristics for each field are given in **Table 1.**

**Table 1:** Soil physicochemical properties of the four different grassland fields.

Field	pH value	Bulk density (g cm^-3^)	Soil organic matter (SOM, g kg^-1^)	Total N (% of DM)	Total C (% of DM)	C/N
Higher Wyke Moor	5.33 ± 0.35 c	0.85 ± 0.04 b	9.30 ± 0.88 a	0.44 ± 0.05 a	3.81 ± 0.52 a	8.73 ± 0.28 a
Longlands North	5.53 ± 0.04 bc	0.91 ± 0.06 ab	6.78 ± 0.41 c	0.43 ± 0.02 a	3.12 ± 0.07 b	7.34 ± 0.20 c
Great Field	5.71 ± 0.19 b	0.97 ± 0.06 a	7.90 ± 0.37 b	0.44 ± 0.02 a	3.52 ± 0.20 ab	7.98 ± 0.21 b
Longlands East	6.15 ± 0.06 a	0.96 ± 0.03 ab	9.35 ± 0.52 a	0.44 ± 0.03 a	3.60 ± 0.32 ab	8.20 ± 0.39 ab

*Data are mean values ± SE (*n* = 6). Significant differences among grassland managements within each variable were tested using Duncan’s multiple range test (*P* < 0.05) and are indicated by different letters.*

### Invertebrate Extraction and Separation

The cores were placed on a Tullgren funnel system (Burkard Manufacturing, Co., Ltd, Rickmansworth, UK; mesh 5 mm) and were collected in a saturated salt solution. The cores were held in the funnels for 14 days; invertebrate collections were sorted, identified and counted under a microscope (Crotty et al., 2014). These were as follows the four main Collembola orders comprised Entomobryomorpha, Poduromorpha, Neelipleona and Symphypleona, the four main Orders of soil dwelling Acari comprising Astigmata, Mesostigmata, Oribatida and Prostigmata, where possible, these were further identified to a higher taxonomic level (Supplementary Table S1). All other invertebrates collected were identified to Order level, apart from the Coleoptera where the majority were identified to Family level – (i.e., Carabidae, Chrysomelidae, Cuculionidae, Elateridae, Ptilidae, and Staphylinidae. Diptera were sorted to Order level apart from Tipulidae larvae which were analysed separately.

Trophic level and Acari/Collembola grouping were determined according to Crotty et al. (2014). Trophic 0 (T0) represents herbivores. Trophic 1 (T1) represents primary decomposers. Trophic 2 (T2) represents secondary decomposers. Trophic 3 (T3) represents micro-predator. Trophic 4 (T4) repre-sents macro-predator. In this study, for the Acari, the primary decomposers (T1) comprised Ixodida (Ixodes) and Oribatida (Astigmata); Secondary decomposers (T2) contained Oribatida (Brachypyline and Macropyline), Prostigmata (Anystina, Hetero-stigmata, Parasitengonina, and Raphignathina); Micro-predators (T3) included the Mesostigmata (Gamasina, Uropodidae). The Collembolan fauna was comprised of the T0 Herbivores Symphypleona (Arrhopalitidae, Bourletiellidae, Dicyrtomidae, Mackenziellidae, Sminthuridae, Sminthurididae, Sphyrothecinae, and Sturmiidae); Primary decomposers (T1) Folsomia and Neelipleona (Neelidae); Secondary decomposers (T2) contained Actaletidae, Entomobryomorpha (Entomobryidae and Isotomidae), Poduromorpha (Brachystomellidae, Hypo gastruridae, Onychiuridae, Poduridae, and Tullbergiidae).

### Data Analysis

Soil faunal taxonomic richness (*S*) was represented by the number of taxonomic groups at each sampling occasion. All data were presented as mean ± standard error, unless otherwise stated. All statistical analyses were performed using SPSS 13.0 (SPSS, Inc., Chicago, IL, USA). Canonical correspondence analysis (CCA; model choice depending on length of first gradient) was performed to analyse the influence of soil management and environmental factors with respect to the soil fauna community composition. Principal component analysis (PCA) was explored to analyze the influence of field management and sample time on soil fauna community structure. Ordination analyses and hypothesis testing were conducted in CANOCO for Windows v. 4.5 (ter Braak and Šmilauer, 2002). The species data matrix used in CCA were derived from the original individual number data. To meet the assumptions of normality and homogeneity, the data were transformed if necessary by arcsine, square root, or log_10_(x+1).

## Results

**FIGURE 2:**
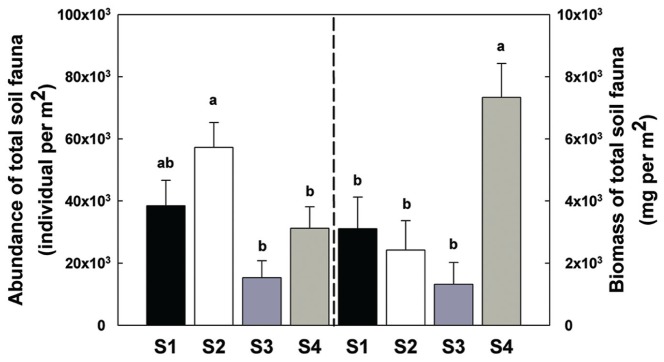
**Abundance and biomass of total soil fauna among sample times.** Bars represent mean values ± SE (*n* = 24 in abundance; *n* = 4 in biomass). Different lowercase letters represent significant differences among sample times (*P* < 0.05) using one way ANOVA test and Duncan’s multiple range test.

**FIGURE 3:**
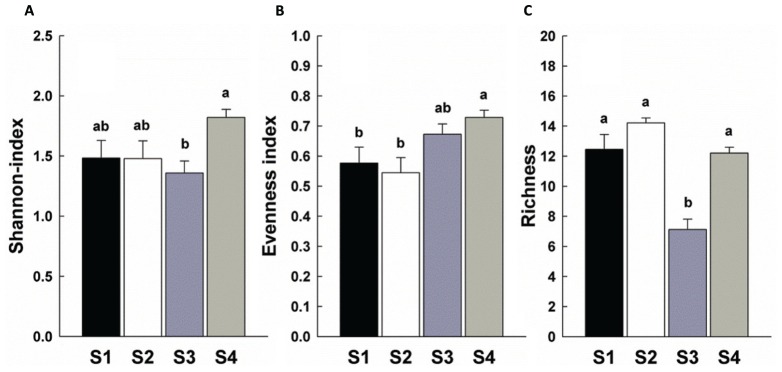
**Diversity indices of soil fauna community among sample times.** Bars represent mean values ± SE (*n* = 24). Different lowercase letters represent significant differences among sample times (*P* < 0.05) using one way ANOVA test and Duncan’s multiple range test. **(A)** Shannon-Weiner index; **(B)** Evenness index; **(C)** Richness.

There was a significant increase in total invertebrate abundance (**Figure 2**) at S2 (*P* < 0.05) followed by a significant reduction post-ploughing (S3, *P* < 0.05) where the lowest abundance was recorded. After 1 year (S4) the total abundance had recovered to pre-treatment levels. Although, the total abundance recovered to previous levels, the total biomass was greater at S4 (**Figure 2**). The Shannon diversity index (H’), Richness (soil fauna Taxon number, *S*) and Evenness index (*E*) were used to analyse impacts of sampling time on diversity of soil fauna communities (**Figure 3**). There was no significant difference in H’ over the first three sampling occasions, but it was significantly (*P* < 0.05) greater than at S4 1 year later post-reseeding. There was a significant (*P* < 0.05) increase in *E* after ploughing which was maintained through to the following year. Taxon richness was greatest after the spray application with a significant (*P* < 0.05) reduction due to ploughing, however there was a significant (*P* < 0.05) recovery over the growth of the reseeded pasture back to pre-ploughing levels.

**FIGURE 4:**
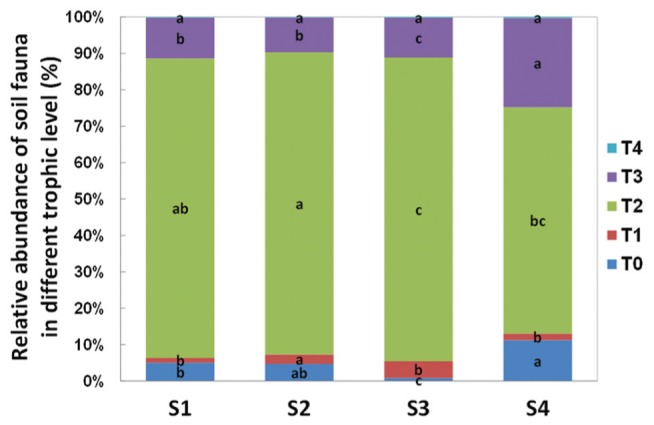
**Relative abundance of soil fauna in different trophic level detected in each sample time.** T0 represent herbivores; T1 represent primary decomposers; T2 represent secondary decomposers; T3 represent micro-predator; T4 represent macro-predator. S1 represent sample time 1, before herbicide spray; S2 represent sample time 2, after spray and before plough; S3 represent sample time 3, after plough and before seed; S4 represent sample time 4, after seed. Bars represent stacked relative abundance ratio (*n* = 24). Different lowercase represent significant differences among sample times of each trophic level (*P* < 0.05) using one way ANOVA test and Duncan’s multiple range test.

In terms of individual number and relative abundance of each trophic level, the secondary decomposers were the most abundant group (**Figure 4**). The micro-predators (T3) were the second most abundant group in this study. At S4, 1 year post-reseed, the proportion of this group was significantly greater than in previous samplings.

**FIGURE 5:**
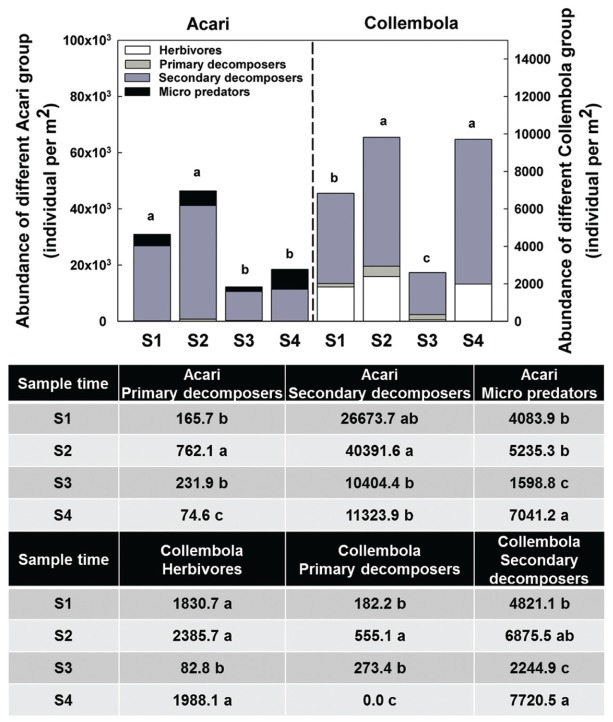
**Abundance of different Acari and Collembola group among sample times.** Bars represent stacked sum values of different Acari/Collembola groups. Bars represent stacked mean values (*n* = 24). Different lowercase letters represent significant differences among sample times (*P* < 0.05) using one way ANOVA test and Duncan’s multiple range test. Divergence analysis was listed in table, different lowercase of each row represented significant difference among sample times in given Acari/Collembola groups.

**FIGURE 6:**
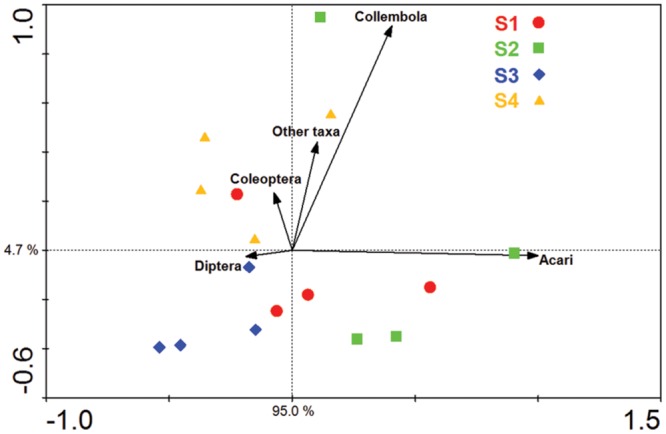
**Principal component analysis (PCA) of the soil fauna community and major soil fauna groups.** S1 represent sample time 1, before herbicide spray; S2 represent sample time 2, after spray and before plough; S3 represent sample time 3, after plough and before seed; S4 represent sample time 4, after seed.

Acari and Collembola were the two most abundant soil fauna groups in our study, making up over 90% of the total soil faunal community. The Collembola population was significantly reduced by ploughing, but had recovered 1 year later (S4). The Acari populations were also reduced by ploughing, but different trophic groups showed different recovery patterns. The decomposer population failed to recover, but the predaceous mites did recover previous population levels (**Figure 5**). PCA analysis of the soil fauna community versus sample times showed a significant effect of sample time on soil fauna community and the different taxonomic groups (**Figure 6**). Most data from S1, S2, and S3 were distributed mainly along the x axis, the S4 was separated from most of points along the y axis. Acari had a positive correlation with S2 and S1, Collembola was positive correlated with S2 and S4.

**FIGURE 7:**
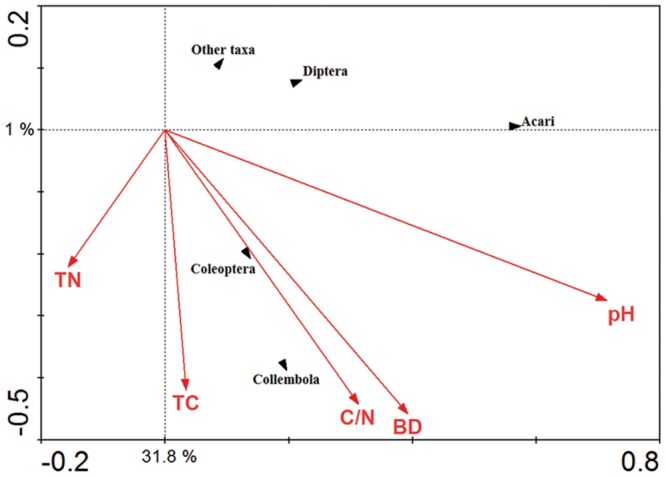
**Canonical correspondence analysis (CCA) of the major soil fauna groups in response to vectors of significant soil chemical properties.** TN represented total nitrogen content; TC represented total carbon content; C/N represented total carbon content/ nitrogen content; BD represented soil bulk density; pH represented soil pH value.

The significance of soil chemical variables in relation to the soil fauna major groups was explored using CCA (**Figure 7**). The Acari were positively correlated with soil pH whilst the Collembola and Coleoptera were positively correlated with soil total carbon content, total C/N ratio and soil bulk density.

## Discussion

Soil invertebrate communities can be affected by fertilization, tillage regimes, or other grassland management (Zhan et al., 2014; Zhu and Zhu, 2015). In this study, the Acari were the numerically dominant soil invertebrates in this study. The Acari covened most trophic groups; primary decomposers, secondary decomposers and micro-predators in this study. This was due to them encompassing a broad range of feeding guilds, including both specialized and polyphagous predators, parasites, herbivores, fungivores, microbivores, detritivores, scavengers, and omnivores (Krantz and Lindquist, 1979; Lindquist, 1979; Walter, 1987). The most abundant groups were the Oribatida and Mesostigmata. The Oribatid Brachychthonioidea were the numerically most abundant and represented between 24.5% (at S4) and 84.5% (at S3) of total soil fauna in this study with a mean value of 58.8% relative abundance. This result was similar to some studies conducted in Canada, which found that the members of Brachychthoniidae dominate the oribatid mite community in fescue grassland of southern Alberta (Clapperton et al., 2002; Osler et al., 2008). The Oribatida have been reported to be one of the most numerically dominant arthropod groups in the organic horizons of most soils (Norton, 1985), and feed on a wide variety of particulate matter including living and dead plant and fungal material, lichens and carrion (Siepel, 1990). The Mesostigmata was the second most dominant Acari group in this study and these have been shown to be the numerically dominant predators in soil and litter of grassland ecosystems (Behan-Pelletier and Kanashiro, 2010; Crotty et al., 2014). Mesostigmata primarily feed on nematodes, Collembola, soft-bodied mites, insect larvae, and small insects, and they respond rapidly to increased prey in the habitat (Behan-Pelletier and Kanashiro, 2010).

Collembola can occupy all the trophic levels in belowground detritus food-webs (Moore et al., 1988) and together with Acari usually account for around 95% of the microarthropods in soils (Seastedt, 1984). Our findings confirmed this and our results showed that plant residue improvement (after herbicide spraying, S2) could lead to an increase in the abundance of the Collembola, possibly due to the fact that, although they can occupy all trophic levels most Collembola tend to be either microphages, feeding on soil microflora, and/or detritivores, scavenging on dead organic matter and plant litter (Bardgett et al., 1993).

At the outset of the study, there had been no gross perturbation for many years and consequently the soil faunal community was in a stable condition. After the herbicide application (S2), the total number of soil invertebrates peaked (approximately 230000 individuals per m^2^). Similar to some previous studies. Greenslade et al. (2010) found that herbicides had negligible effects on ants and springtails in an Australian wheat field due to having less exposure in surface soil and hydrophobic structure of fauna body. Lins et al. (2007) pointed out that Acari and Collembola could use herbicides to breed themselves until the decomposition product occurs.

**FIGURE 8:**
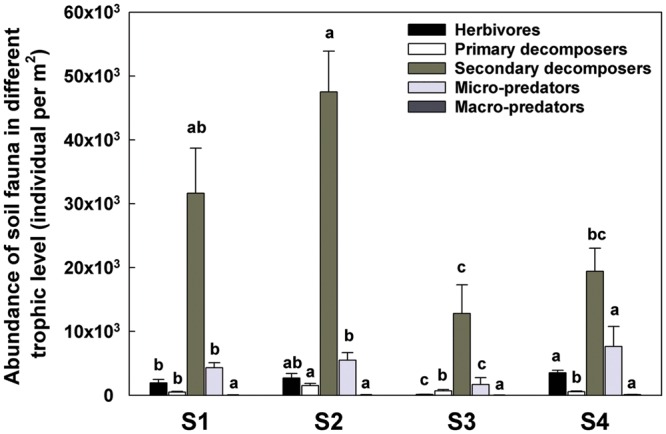
**Abundance of soil fauna in different trophic level detected in each sample time.** T0 represent herbivores; T1 represent primary decomposers; T2 represent secondary decomposers; T3 represent micro-predator; T4 represent macro-predator. S1 represent sample time 1, before herbicide spray; S2 represent sample time 2, after spray and before plough; S3 represent sample time 3, after plough and before seed; S4 represent sample time 4, after seed. Bars represent stacked relative abundance ratio (*n* = 24). Different lowercase represent significant differences among sample times of each trophic level (*P* < 0.05) using one way ANOVA test and Duncan’s multiple range test.

The increasing amount of dead material favored the oribatid mites Brachypyline and Macropyline and the Collembola all of which are among the most important decomposers and provide food source for other soil invertebrates (Norton, 1985; Siepel, 1990). The comminution of the dead plant material by these organisms can impact on the habitat in ways that facilitate microbial activity (Eisenhauer et al., 2007). The increased numbers of herbivores and decomposers, also promoted the abundance of some micro-predators such as Mesostigmatid mites (Supplementary Table S1; **Figure 8**).

After ploughing (S3), there were significant reductions of soil invertebrate individual number and biomass. Total soil invertebrate number decreased from approximately 23000 to 61000 individuals per m^2^ and total arthropod biomass decreased from 9680 to 5280 mg per m^2^. This could be attributed to the greater impact of ploughing on the larger arthropods such as the Coleoptera and Diptera. Both mites and Collembola have been used as bioindicators of soil quality due to their high sensitivity to disturbances (Prasse, 1985; Hopkin, 1997), and both these groups declined sharply in number with ploughing. Ploughing influences the distribution of resources. Thus, the fauna in the deeper horizon is enriched by a surviving fraction of the surface fauna which to some extent compensates for the mortality due to abrasion, etc. On the other hand, the less abundant fauna of deeper soil layers are translocated to the surface layers where they are more exposed to drought and other microclimatic extremes (Petersen, 2002).

After reseeding (S4), and after all fields had had 1 year of recovery, the total soil invertebrate number and biomass increased (total number from approximately 61300 in S3 to 125000 individuals per m^2^ in S4; total biomass from 5280 in S3 to 29300 mg per m^2^ in S4). Here, the Collembola populations recovered to their pre-treatment levels. Although, the Acari populations appeared to recover, when broken down the decomposer populations remained low, but the predatory mites significantly increased showing a significant increase in the predator/prey ratio at this time point (**Figure 4** and Supplementary Table S2), indicating the relative instability of the community.

The CCA indicates that soil invertebrate groups as Acari, Collembola, Coleoptera, Diptera, and other taxa are influenced by the soil pH value, bulk density, soil total carbon and nitrogen content, and total C/N ratio. Similar results have been reported by other authors, who concluded that a pH close to neutral is optimal for Acari (Bedano et al., 2006). Soil total carbon content, total C/N ratio and bulk density showed a strong relationship with arthropod groups such as Collembola and Coleoptera. Consistent with some previous studies from Europe (Brennan et al., 2006; García et al., 2010).

## Conclusion

Herbicide application tended to increase soil invertebrate abundance due to the plant residue improvement, less exposure and edibility of herbicide for some soil arthropod. Whereas subsequent ploughing significantly reduced soil invertebrate number and biomass due to its disturbance effects. Ploughing had an obvious negative effect on soil primary and secondary decomposers. This change was mainly due to the Acari (especially Oribatida) and some Coleoptera group abundance variations. Reseeding also reduced individual numbers in groups, and favored those predators with larger body size and individual weight. Over the following year, different arthropod groups responded differently, with Collembola populations recovering to pre-treatment levels. However, the Acari populations still appeared to be in flux 1 year on.

Wagg et al. (2014) showed that loss of soil biodiversity together with the simplification of communities negatively impacts on many ecosystem functions. Thus, maintenance of a healthy soil food web is key to maintaining and in increasing agricultural productivity (Crotty et al., 2015). This study highlights how common agricultural practices impact on soil fauna communities, this impact might cause some ecological function change of soil, and how different components of the community respond differently to the disturbances caused.

## Author Contributions

PM and JZ conceptualized the study, WL collected, processed, and identified samples with help from SN. WL primarily interpreted the data with contributions from PM and JZ. WL and PM wrote the manuscript and all authors were involved in reviewing, revision and final approval of the manuscript.

## Conflict of Interest Statement

The authors declare that the research was conducted in the absence of any commercial or financial relationships that could be construed as a potential conflict of interest.
