# Supplementary material for: Impact of Grassland Reseeding, Herbicide Spraying and Ploughing on Diversity and Abundance of Soil Arthropods
Source: Front Plant Sci. 2016 Aug 9;7:1200. doi: 10.3389/fpls.2016.01200 (PMC4977627; doi:10.3389/fpls.2016.01200)
Supplement: Supplementary file 1 [file Table_1.DOC]

Table S1 Detail list of soil fauna taxa composition, abundance of each sampling time and field type.

Table S2 Detail list of soil fauna taxa biomass of each sampling time and field type.

Table S1

**(a)**

| Sample 1 | Number per m^2^ | | | |
| --- | --- | --- | --- | --- |
|  | Great field | Higher Wyke Moor | Longlands North | Longlands East |
| Acari: Ixodida: Ixodes | — | 166 (±109) | — | 199 (±186) |
| Acari: Mesostigmata: Gamasina | 3810 (±1639) | 3645 (±1532) | 2485 (±542) | 6130 (±3453) |
| Acari: Mesostigmata: Uropodidae | — | 199 (±186) | 33 | 33 |
| Acari: Oribatida: Astigmata | — | 199 (± 222) | 66 (± 57) | 33 |
| Acari: Oribatida: Brachypyline + Macropyline | 25381 (±7151) | 11233 (± 6386) | 20444 (±9694) | 45030 (±22621) |
| Acari: Prostigmata Raphignathina | — | 66 | — | — |
| Acari: Prostigmata: Anystina | 66 | — | — | — |
| Acari: Prostigmata: Heterostigmata | 729 (±194) | 895 (±1009) | 1292 (±946) | 961(±462) |
| Acari: Prostigmata: Parasitengonina | 99 | 99 (±61) | 33 | 364 (±237) |
| Araneae: Steatoda | 33 | 33 | 33 | 33 |
| Blattodea: Periplaneta | — | — | — | 33 |
| Coleoptera | — | 33 | — | — |
| Coleoptera larva | 99 | — | — | — |
| Coleoptera: Cantharidae larva | — | 33 | — | 66 (±57) |
| Coleoptera: Carabidae larva | 66 | 66 (±57) | — | — |
| Coleoptera: Elateridae | — | — | — | 33 |
| Coleoptera: Endomychidae | — | — | — | — |
| Coleoptera: Galerucinae larva | 33 | — | — | — |
| Coleoptera: Staphylinidae larva | 66 (±57) | 133 (±91) | 66 (±57) | 199 (±140) |
| Coleoptera: Tenebrionidae | 33 | — | — | — |
| Collembola: Actaletidae | — | 33 | — | — |
| Collembola: Entomobryomorpha: Entomobryidae | 2087 (±1261) | 2750 (±1370) | 1557 (±1714) | 828 (±301) |
| Collembola: Entomobryomorpha: Isotomidae | 1491 (±500) | 2883 (±974) | 431 (±332) | 2783 (±2891) |
| Collembola: Folsomia | 33 | — | — | 132 |
| Collembola: Neelipleona: Neelidae | 33 | 66 | 298 (±304) | 166 (±130) |
| Collembola: Poduromorpha: Brachystomellidae | 66 (±57) | 298 (±260) | — | 166 (±109) |
| Collembola: Poduromorpha: Hypogastruridae | 66 (±57) | 232 (±147) | 33 | 298 (±153) |
| Collembola: Poduromorpha: Onychiuridae | — | 132 (±91) | 66 (±57) | 33 |
| Collembola: Poduromorpha: Tullbergiidae | 497 (±335) | 265 (±152) | 1358 (±1145) | 961 (±513) |
| Collembola: Symphypleona: Arrhopalitidae | — | 66 | 33 | — |
| Collembola: Symphypleona: Bourletiellidae | 66 (±57) | 298 (±278) | — | 33 |
| Collembola: Symphypleona: Dicyrtomidae | 232 (±130) | 497 (±335) | 132 (±115) | — |
| Collembola: Symphypleona: Mackenziellidae | — | 33 | — | 33 |
| Collembola: Symphypleona: Sminthuridae | 99 (±93) | 331 (±229) | 33 | 99 (±93) |
| Collembola: Symphypleona: Sminthurididae | 1093 (±738) | 828 (±609) | 497 (±414) | 2783 (±2273) |
| Collembola: Symphypleona: Sphyrothecinae | — | 99 (±93) | 33 | — |
| Collembola: Symphypleona: Sturmiidae | — | — | 66 | — |
| Dermaptera | 33 | 33 | — | — |
| Diptera Larva | 33 | — | — | 33 |
| Diptera: Cecidomyiidae larva | — | 33 | — | 66 (±57) |
| Diptera: Chironomidae | — | — | — | 33 |
| Diptera: Sciaridae | 66 (±57) | — | — | 33 |
| Diptera: Trichoceridae | 66 (±57) | 33 | — | — |
| Diptora: Cyclorrhapha | — | — | 66 (±57) | 33 |
| Haplotaxida: Enchytraeidae | 99 (±61) | 33 | 33 | 33 |
| Hemiptera: Delphacidae | 33 | — | — | — |
| Hymenoptera: Chalcidoidea | 66 (±57) | — | — | — |
| Hymenoptera: Ichneumonoidea | 33 | — | — | — |
| Juvenile earthworm | — | 99 (±61) | 33 | 66 (±57) |
| Psocoptera | — | — | 33 | — |
| Thysanoptera | 33 | — | 66 (±57) | 331 (±134) |
| **Total Groups** | **32** | **33** | **25** | **32** |
| **Σ** | **36806** | **25843** | **29154** | **62057** |

**(b)**

| Sample 2 | Number per m^2^ | | | |
| --- | --- | --- | --- | --- |
|  | Great Field | Higher Wyke Moor | Longlands North | Longlands East |
| Acari: Ixodida: Ixodes | 132 (±134) | — | 464 (±303) | 729 (±524) |
| Acari: Mesostigmata: Gamasina | 4274 (±1237) | 6693 (±2673) | 2319 (±474) | 7555 (±995) |
| Acari: Mesostigmata: Uropodidae | 66 (±57) | 33 | — | — |
| Acari: Oribatida: Astigmata | 298 (±219) | 464 (±250) | 33 | 928 (±1215) |
| Acari: Oribatida: Brachypyline + Macropyline | 32671 (±5831) | 22432 (±8566) | 41949 (±15276) | 55866 (±10085) |
| Acari: Prostigmata: Heterostigmata | 1657 (±479) | 1425 (±309) | 1856 (±626) | 2982 (±1275) |
| Acari: Prostigmata: Parasitengonina | 298 (±116) | 199 (±172) | 33 | 199 (±222) |
| Aphids (Hemiptera: Aphidoidea) | — | — | — | 199 (±186) |
| Araneae: Steatoda | 199(±222) | — | 33 | — |
| Blattodea: Periplaneta | — | 33 | — | — |
| Chilopoda: Centipede | — | 33 | — | — |
| Coleoptera | — | 33 | — | — |
| Coleoptera larva | — | — | — | 33 |
| Coleoptera: Cantharidae larva | — | 33 | 66 (±57) | 66 (±57) |
| Coleoptera: Carabidae larva | — | 33 | — | 66 |
| Coleoptera: Galerucinae larva | — | — | 132 (±115) | — |
| Coleoptera: Staphylinidae larva | 99 (±61) | 33 | 33 | 199 (±140) |
| Collembola: Entomobryomorpha: Entomobryidae | 762 (±513) | 5799 (±1264) | 431 (±237) | 994 (±803) |
| Collembola: Entomobryomorpha: Isotomidae | 2386 (±557) | 7720 (±2097) | 961 (±727) | 1657 (±1461) |
| Collembola: Folsomia | 66 | 66 (±57) | 1027 (±1351) | 431 (±215) |
| Collembola: Neelipleona: Neelidae | 99 (±61) | 166 (±147) | 199 (±121) | 166 (±109) |
| Collembola: Poduromorpha: Brachystomellidae | — | 132 (±134) | 33 | 232 (±226) |
| Collembola: Poduromorpha: Hypogastruridae | 66 (±57) | 630 (±478) | 199 (±172) | 3214 (±3705) |
| Collembola: Poduromorpha: Onychiuridae | — | 33 | — | 33 |
| Collembola: Poduromorpha: Poduridae | 33 | — | — | — |
| Collembola: Poduromorpha: Tullbergiidae | 563 (±301) | 398 (±233) | 563 (±332) | 663 (±537) |
| Collembola: Symphypleona: Bourletiellidae | 66 (±57) | 99 (±93) | 33 | — |
| Collembola: Symphypleona: Dicyrtomidae | — | 99 (±93) | — | — |
| Collembola: Symphypleona: Mackenziellidae | — | 33 | — | — |
| Collembola: Symphypleona: Sminthuridae | 298 (±182) | 895 (±327) | 398 (±140) | 596 (±298) |
| Collembola: Symphypleona: Sminthurididae | 464 (±269) | 1491 (±490) | 1822 (±826) | 3247 (±1059) |
| Dermaptera | 33 | — | — | — |
| Diptera Larva | — | 33 | 33 | — |
| Diptera: Sciaridae | 33 | 99 (±93) | 99 (±93) | 33 |
| Diptera: Trichoceridae | — | 33 | — | — |
| Diptora: Cyclorrhapha | 66 (±57) | — | 132 (±134) | — |
| Haplotaxida: Enchytraeidae | 99 (±61) | 66 (±57) | — | 33 |
| Hemiptera: Delphacidae | 33 | 33 | — | — |
| Hymenoptera: Chalcidoidea | 99 (±61) | — | 33 | 33 |
| Juvenile earthworm | 33 | 33 | 66 (±57) | 66 (±57) |
| Phthiraptera: Anoplura | 33 | 99 (±93) | — | — |
| Psocoptera | — | 132 | — | — |
| Thysanoptera | 166 (±130) | 66 (±57) | 265 (±57) | 497 (±395) |
| **Total Groups** | **29** | **35** | **27** | **26** |
| **Σ** | **45191** | **49765** | **53344** | **80717** |

**(c)**

| Sample 3 | Number per m^2^ | | | |
| --- | --- | --- | --- | --- |
|  | Great field | Higher Wyke Moor | Longlands North | Longlands East |
| Acari: Ixodida: Ixodes | — | 166 | — | 132 (±134) |
| Acari: Mesostigmata: Gamasina | 1292 (±758) | 166 (±130) | 166 (±109) | 4771 (±4471) |
| Acari: Oribatida: Astigmata | 166 | 33 | 33 | 398 (±444) |
| Acari: Oribatida: Brachypyline + Macropyline | 18125 (±12265) | 2154 (±837) | 5732 (±1599) | 12359 (±4873) |
| Acari: Prostigmata: Heterostigmata | 961 (±1011) | 199 (±172) | 696 (±519) | 1392(±824) |
| Aphids (Hemiptera: Aphidoidea) | — | — | — | — |
| Araneae: Steatoda | — | 66 | — | — |
| Coleoptera: Cantharidae larva | 66 (±57) | — | 33 | — |
| Coleoptera: Coccinellidae | 33 | — | — | — |
| Coleoptera: Elateridae | — | — | — | — |
| Coleoptera: Lathridiidae | — | — | 33 | — |
| Coleoptera: Staphylinidae larva | — | 99 | — | — |
| Collembola: Entomobryomorpha: Entomobryidae | 33 | — | 99 (±61) | 663 (±382) |
| Collembola: Entomobryomorpha: Isotomidae | 232 (±109) | 99 (±93) | 132 (±115) | 596 (±365) |
| Collembola: Folsomia | 232 | 66 (±57) | 199 (±222) | 530 (±250) |
| Collembola: Neelipleona: Neelidae | — | 66 (±57) | — | — |
| Collembola: Poduromorpha: Brachystomellidae | — | 66 (±57) | — | 166 (±109) |
| Collembola: Poduromorpha: Hypogastruridae | 364 (±446) | 132 (±57) | — | 1093 (±867) |
| Collembola: Poduromorpha: Tullbergiidae | 1822 (±1859) | 431 (±332) | 398 (±306) | 2651 (±1268) |
| Collembola: Symphypleona: Sminthuridae | 66 (±57) | — | — | — |
| Collembola: Symphypleona: Sminthurididae | 66 (±57) | — | 66 | 132 (±134) |
| Diptera Larva | 398 | 99 (±93) | 66 | 99 (±61) |
| Diptera: Cecidomyiidae larva | — | — | — | 33 |
| Diptera: Sciaridae | — | — | — | — |
| Diptora: Cyclorrhapha | — | 99 (±61) | 66 (±57) | 33 |
| Haplotaxida: Enchytraeidae | — | 33 | — | — |
| Hymenoptera: Chalcidoidea | — | — | 33 | 66 |
| Hymenoptera: Ichneumonoidea | — | — | — | — |
| Juvenile earthworm | — | — | 33 | — |
| Lepidoptera: Brimstone moth | — | 33 | 66 (±57) | — |
| Phthiraptera: Anoplura | — | 33 | — | — |
| Thysanoptera | 33 | — | 33 | 33 |
| **Total Groups** | **16** | **18** | **18** | **18** |
| **Σ** | **23988** | **4040** | **7917** | **25379** |

**(d)**

| Sample 4 | Number per m^2^ | | | |
| --- | --- | --- | --- | --- |
|  | Great field | Higher Wyke Moor | Longlands North | Longlands East |
| Acari: Ixodida: Ixodes | — | — | — | — |
| Acari: Mesostigmata: Gamasina | 3446 (±474) | 2916 (±1040) | 5169 (±1187) | 12658 (±3519) |
| Acari: Mesostigmata: Uropodidae | 331 (±152) | 99 (±93) | 132 (±115) | 3413 (±3866) |
| Acari: Oribatida: Astigmata | — | 33 | — | 265 (±259) |
| Acari: Oribatida: Brachypyline + Macropyline | 5103 (±997) | 6494 (±2560) | 13751 (±5068) | 16700 (±2442) |
| Acari: Prostigmata: Heterostigmata | 928 (±295) | 762 (±266) | 961 (±609) | 596 (±378) |
| Aphids (Hemiptera: Aphidoidea) | 33 | 66 (±57) | — | 265 (±181) |
| Araneae: Steatoda | 66 | 33 | — | 99 (±93) |
| Chilopoda: Centipede | 132 (±57) | 33 | — | — |
| Coleoptera: Cantharidae larva | 33 | 66 (±57) | 33 | 33 |
| Coleoptera: Carabidae | — | — | — | — |
| Coleoptera: Carabidae larva | 99 | — | — | — |
| Coleoptera: Staphylinidae | — | — | — | — |
| Coleoptera: Staphylinidae larva | 696 (±565) | 132 (±57) | 530 (±167) | 795 (±351) |
| Collembola: Entomobryomorpha: Isotomidae | 2154 (±1639) | 994 (±695) | 1657 (±683) | 3545 (±1379) |
| Collembola: Poduromorpha: Hypogastruridae | 1789 (±1048) | 3910 (±1660) | 2319 (±490) | 6395 (±1132) |
| Collembola: Poduromorpha: Tullbergiidae | 1756 (±1437) | 3512 (±2530) | 1657 (±1491) | 1193 (±476) |
| Collembola: Symphypleona: Sminthurididae | 2319 (±1113) | 1955 (±731) | 1292 (±701) | 2386 (±658) |
| Diptera: Cecidomyiidae larva | 298 (±116) | 464 (±134) | 232 (±130) | 331 (±152) |
| Diptera: Sciaridae | 166 (±83) | 331 (±250) | 364 (±191) | 398 (±140) |
| Haplotaxida: Tubificid | 33 | 132 | — | — |
| Juvenile earthworm | 99 (±61) | 99 (±61) | 132 (±57) | 99 (±61) |
| Lepidoptera: Pieridae | 33 | — | — | — |
| Thysanoptera | 1259 (±708) | 663 (±319) | 1856 (±853) | 1922 (±547) |
| **Total Groups** | **21** | **20** | **15** | **18** |
| **Σ** | **20806** | **22727** | **30118** | **51159** |

Note: Data presented as mean ± standard error (n=6).

Table S2

**(a)**

| Sample 1 | Biomass per m^2^ (mg) | | | |
| --- | --- | --- | --- | --- |
|  | Great field | Higher Wyke Moor | Longlands North | Longlands East |
| Acari: Ixodida: Ixodes | 10.74 | 15.31 | 14.12 | 61.83 |
| Acari: Mesostigmata | 134.79 | 102.78 | 94.04 | 128.03 |
| Acari: Oribatida | 75.95 | 18.69 | 46.72 | 86.48 |
| Acari: Prostigmata | 31.41 | 4.57 | 11.53 | 42.74 |
| Araneae: Steatoda | — | — | 2.78 | 1.39 |
| Coleoptera larva | 12.53 | 14.51 | 8.75 | — |
| Coleoptera: Ptilidae | 30.42 | 37.97 | — | — |
| Coleoptera: Carabidae | — | — | — | — |
| Coleoptera: Carabidae larva | 3.78 | — | — | — |
| Coleoptera: Staphylinidae | — | — | 1.99 | 94.63 |
| Coleoptera: Staphylinidae larva | 6.36 | 37.77 | — | — |
| Collembola: Entomobryomorpha | 20.08 | 90.06 | 16.70 | 27.24 |
| Collembola: Neelipleona | — | — | 2.19 | — |
| Collembola: Poduromorpha | 26.84 | 8.95 | 18.49 | 10.54 |
| Collembola: Symphypleona | 18.69 | 13.12 | 6.76 | 4.18 |
| Diptera | 5.57 | — | 6.36 | 1.99 |
| Diptera: Cecidomyiidae larva | — | 1102.20 | — | 746.33 |
| Diptera: Sciaridae | — | — | — | 17.89 |
| Haplotaxida: Enchytraeidae | 23.66 | — | 4.77 | — |
| Hymenoptera | 111.53 | — | 4.97 | — |
| Juvenile earthworm | — | 3950.16 | 2520.12 | 2491.88 |
| Phthiraptera: Anoplura | — | 6.56 | — | 2.58 |
| Thysanoptera | 10.14 | — | 7.95 | 12.53 |
| **Total Groups** | **16** | **13** | **16** | **15** |
| **Σ** | **525.47** | **5402.65** | **2768.24** | **3730.26** |

**(b)**

| Sample 2 | Biomass per m^2^ (mg) | | | |
| --- | --- | --- | --- | --- |
|  | Great field | Higher Wyke Moor | Longlands North | Longlands East |
| Acari: Ixodida: Ixodes | 38.77 | — | 80.12 | 84.10 |
| Acari: Mesostigmata | 252.49 | 271.97 | 150.30 | 120.08 |
| Acari: Oribatida | 40.35 | 17.10 | 11.93 | 25.05 |
| Acari: Prostigmata | 1.59 | 25.45 | 11.93 | 12.13 |
| Aphids (Hemiptera: Aphidoidea) | 3.58 | 13.12 | — | 23.46 |
| Araneae: Steatoda | 36.78 | — | — | — |
| Coleoptera: Staphylinidae | 7.95 | — | — | — |
| Coleoptera: Staphylinidae larva | 67.20 | 328.63 | 13.12 | 237.38 |
| Collembola: Entomobryomorpha | 29.42 | 90.66 | 14.71 | 25.85 |
| Collembola: Poduromorpha | 16.50 | 39.37 | 19.48 | 16.50 |
| Collembola: Symphypleona | 7.36 | 22.47 | 16.90 | 7.75 |
| Diptera: Cecidomyiidae larva | — | 17.30 | 11.73 | — |
| Diptera: Sciaridae | 43.94 | 16.90 | 175.35 | 4.77 |
| Juvenile earthworm | 318.89 | 123.06 | 4364.28 | 2371.41 |
| Thysanoptera | 16.90 | 4.57 | 6.36 | 4.77 |
| **Total Groups** | **15** | **13** | **13** | **12** |
| **Σ** | **891.86** | **976.56** | **4878.79** | **2933.25** |

**(c)**

| Sample 3 | Biomass per m^2^ (mg) | | | |
| --- | --- | --- | --- | --- |
|  | Great field | Higher Wyke Moor | Longlands North | Longlands East |
| Acari: Ixodida: Ixodes | — | 21.07 | — | 19.68 |
| Acari: Mesostigmata | 24.06 | 16.70 | 17.50 | 13.32 |
| Acari: Oribatida | 12.52 | 3.18 | 4.97 | 2.98 |
| Acari: Prostigmata | 3.78 | 3.98 | 4.97 | 1.39 |
| Aphids (Hemiptera: Aphidoidea) | — | 5.17 | — | — |
| Araneae: Steatoda | — | 4.57 | — | — |
| Coleoptera: Carabidae | — | 379.73 | — | — |
| Coleoptera: Corylophidae | — | — | 74.35 | — |
| Coleoptera: Hydrophilidae | 65.01 | — | — | — |
| Coleoptera: Staphylinidae larva | 10.54 | — | 7.36 | 1.99 |
| Collembola: Entomobryomorpha | 5.96 | 3.58 | 2.78 | 10.14 |
| Collembola: Poduromorpha | 11.13 | 6.96 | 7.36 | 9.54 |
| Collembola: Symphypleona | 4.18 | 3.38 | — | 1.19 |
| Diptera: Cecidomyiidae larva | 23.06 | 18.69 | 15.11 | 27.63 |
| Diptera: Sciaridae | — | 415.71 | 367.01 | 160.44 |
| Haplotaxida: Enchytraeidae | — | 3.38 | — | — |
| Juvenile earthworm | — | — | 1514.34 | — |
| Lepidoptera: Pieridae | — | 814.13 | 1120.29 | — |
| Thysanoptera | 3.38 | — | 3.18 | 1.79 |
| **Total Groups** | **11** | **14** | **13** | **12** |
| **Σ** | **168.79** | **1700.23** | **3147.37** | **266.19** |

**(d)**

| Sample 4 | Biomass per m^2^ (mg) | | | |
| --- | --- | --- | --- | --- |
|  | Great field | Higher Wyke Moor | Longlands North | Longlands East |
| Acari: Ixodida: Ixodes | — | — | — | — |
| Acari: Mesostigmata | 258.25 | 110.93 | 216.90 | 759.25 |
| Acari: Oribatida | 44.93 | 24.46 | 45.53 | 45.52 |
| Acari: Prostigmata | 14.31 | 9.15 | 7.75 | 5.37 |
| Aphids (Hemiptera: Aphidoidea) | 10.54 | 30.82 | — | 21.27 |
| Araneae: Steatoda | 10.14 | 24.45 | — | 39.56 |
| Chilopoda: Centipede | 42.55 | 395.23 | — | — |
| Coleoptera: Cantharidae | 428.83 | 925.26 | 18.69 | 464.42 |
| Coleoptera: Carabidae | — | — | — | — |
| Coleoptera: Carabidae larva | 51.29 | — | — | — |
| Coleoptera: Staphylinidae | — | — | — | — |
| Coleoptera: Staphylinidae larva | 47.32 | 104.77 | 375.75 | 161.04 |
| Collembola: Entomobryomorpha | 93.04 | 11.53 | 103.58 | 63.22 |
| Collembola: Poduromorpha | 30.62 | 98.41 | 98.41 | 82.51 |
| Collembola: Symphypleona | 11.93 | 11.53 | 8.95 | 17.69 |
| Diptera: Cecidomyiidae larva | 76.74 | 82.70 | 135.79 | 124.06 |
| Diptera: Sciaridae | 343.34 | 61.43 | 194.04 | 321.48 |
| Haplotaxida: Tubificid | 70.98 | 105.17 | — | — |
| Juvenile earthworm | 3473.61 | 4125.71 | 8798.14 | 2920.92 |
| Lepidoptera: Pieridae | 2998.85 | — | — | — |
| Thysanoptera | 41.75 | 15.90 | 41.75 | 54.47 |
| **Total Groups** | **19** | **17** | **13** | **15** |
| **Σ** | **8052.8** | **6140.83** | **10051.44** | **5088.14** |
